# Research gaps in the neurodevelopmental assessment of children with complex congenital heart defects: a scoping review

**DOI:** 10.3389/fped.2024.1340495

**Published:** 2024-05-23

**Authors:** Johannes Hofer, Marina Blum, Regina Wiltsche, Nikoletta Deluggi, Daniel Holzinger, Johannes Fellinger, Gerald Tulzer, Gina Blum, Raphael Oberhuber

**Affiliations:** ^1^ Research Institute for Developmental Medicine, Johannes Kepler University of Linz, Linz, Austria; ^2^ Institute of Neurology of Senses and Language, Hospital of St. John of God, Linz, Austria; ^3^ Institute of Linguistics, University of Graz, Graz, Austria; ^4^ Division of Social Psychiatry, University Clinic for Psychiatry and Psychotherapy, Medical University of Vienna, Vienna, Austria; ^5^ Department of Pediatric Cardiology, Children’s Heart Center Linz, Kepler University Hospital, Linz, Austria; ^6^ Department of Inclusive Education, University of Education Upper Austria, Linz, Austria

**Keywords:** complex congenital heart defects (CHD), neurodevelopment, social communication, speech motor, malleable predictors, assessment

## Abstract

**Background:**

Children with congenital heart defects (CHD) are at risk for a range of developmental disabilities that challenge cognition, executive functioning, self-regulation, communication, social-emotional functioning, and motor skills. Ongoing developmental surveillance is therefore key to maximizing neurodevelopmental outcome opportunities. It is crucial that the measures used cover the spectrum of neurodevelopmental domains relevant to capturing possible predictors and malleable factors of child development.

**Objectives:**

This work aimed to synthesize the literature on neurodevelopmental measures and the corresponding developmental domains assessed in children aged 1−8 years with complex CHD.

**Methods:**

PubMed was searched for terms relating to psycho-social, cognitive and linguistic-communicative outcomes in children with CHD. 1,380 papers with a focus on complex CHD that reported neurodevelopmental assessments were identified; ultimately, data from 78 articles that used standardized neurodevelopmental assessment tools were extracted.

**Results:**

Thirty-nine (50%) of these excluded children with syndromes, and 9 (12%) excluded children with disorders of intellectual development. 10% of the studies were longitudinal. The neurodevelopmental domains addressed by the methods used were: 53% cognition, 16% psychosocial functioning, 18% language/communication/speech production, and 13% motor development-associated constructs.

**Conclusions:**

Data on social communication, expressive and receptive language, speech motor, and motor function are underrepresented. There is a lack of research into everyday use of language and into measures assessing language and communication early in life. Overall, longitudinal studies are required that include communication measures and their interrelations with other developmental domains.

## Introduction

One in 100 newborns is affected by a congenital heart defect (CHD) (1). Twenty-five percent of them have a severe CHD that requires early corrective heart surgery within the first year of life (1). In about one third of these severely affected children, a genetic-syndromic disease causes the heart defect(s) (2). Since surgical and cardiological pediatric therapy protocols have improved over the last few years, around 80%–90% of children with CHD now survive to adulthood (3, 4).

Children with CHD are at risk for a range of developmental disabilities that challenge cognition, executive functioning, self-regulation, communication, social-emotional functioning, and motor skills (5–8). Studies have reported a prevalence of learning disability in 20% of children (9, 10), autism spectrum disorder in up to 10% (9, 11, 12), ADHD in up to 5% (13) and visual impairment in around 5% (14).

Despite the extensive knowledge gained about the developmental profiles of children with CHD, little is known about predictive and moderating developmental factors related to quality of life and psychosocial well-being. While only 5% of the variance in cognitive outcomes can be explained by surgical factors (15–17) and 1% by the choice of a cardiopulmonary bypass (18), up to 33% is determined by innate patient- and family-related variables (19, 20). Numerous studies have shown that the total length of stay in hospital is another predictor of cognitive outcome (21). However, with regard to developmental trajectories, a large proportion of the variance currently remains unexplained. There is great diversity in children with CHD, for instance, in the type of heart defect and its pathophysiological consequences, the palliation needed and its cardiocirculatory consequences, the variety of etiologies and the family resources available.

In 2012, the American Heart Association/American Academy of Pediatrics (5) highlighted the increased developmental risk for children with CHD and the need for ongoing developmental surveillance to maximize neurodevelopmental outcome opportunities. In 2020, the Cardiac Neurodevelopmental Outcome Collaborative established a consensus-based, standardized battery for the content and timing of neurodevelopmental assessments for children with complex CHD with the goal of promoting consistent neurodevelopmental care and quality improvement (22, 23). The recommendations include core and extended versions of age-specific assessment batteries.

This scoping review aims to provide a concise snapshot of the measures used to investigate neurodevelopmental domains of children with complex CHD between the ages of one and eight years. The research question was: To what extent do the standardized neurodevelopmental assessment tools cover the spectrum of neurodevelopmental domains relevant for capturing possible predictors and malleable factors of child development?

## Materials and methods

The methodological framework by Arksey and O'Malley (24) was used for this scoping review: (1) identify the research question; (2) identify relevant studies; (3) select studies; (4) chart the data; and (5) collect, summarize and report results.

The data synthesis followed the Preferred Reporting Items for Systematic Reviews and Meta-Analyses (PRISMA) guidelines.

Studies were identified by searching the electronic database PubMed. Initial searches were conducted to identify relevant literature. Each search used the keywords “congenital heart disease” and one or more of the keywords from the following list: “mental health,” “quality of life,” “psychosocial outcome,” “neurodevelopmental outcome,” “social communication,” “parent-child interaction,” “language,” “social cognition,” “family,” “self-esteem,” “anxiety,” “depression”, “functional outcome.”

The quality of the studies was assessed using an adapted version of the Newcastle-Ottawa Scale (25) with a maximum score of 7 points. One point was allocated to each of the following categories:

a) representativeness of the sample (e.g., all syndromes were included, no exclusion due to ethnicity, language or cognitive function);
b) sample size (adequate);
c) adequate control group available;
d) clear definition of the neurodevelopmental measures used;
e) clear description of the domains assessed
f) the study reported on outcomes; and
g) appropriate statistical analysis was conducted and included.

Original articles were included in the review if all of the following criteria were met: (i) study participants were diagnosed with complex CHD and (ii) children aged between 1 and 8 years were included; (iii) the article was in English, peer-reviewed and from the period 1980–2021; (iv) standardized quantitative methods were used to evaluate neurodevelopment; and (v) the study had a fair to good quality [adapted Newcastle-Ottawa Scale (NOS) score >2]. Studies based exclusively on questionnaires or interviews without direct assessment were excluded.

**Figure 1:**
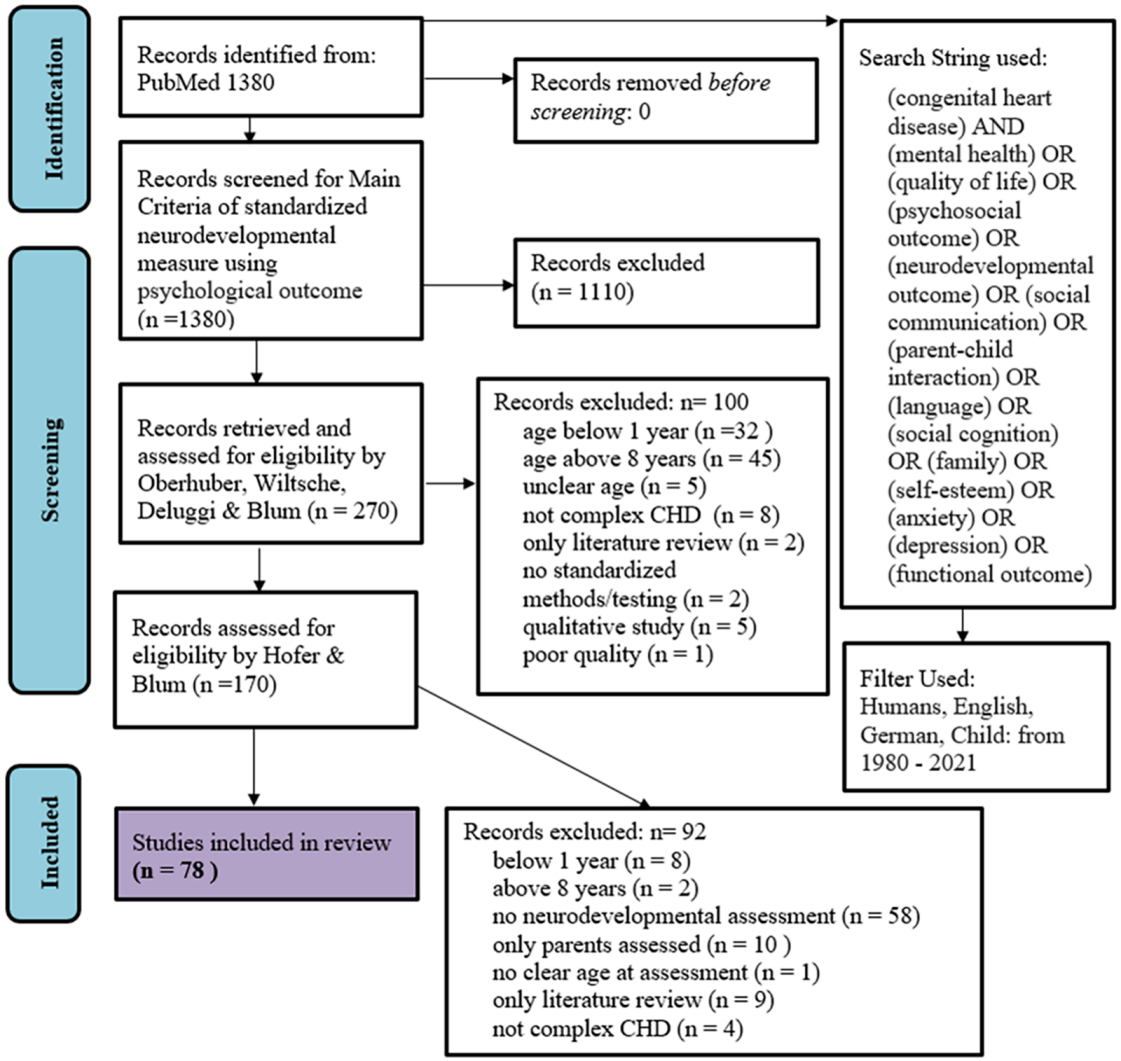
PRISMA 2020 flow chart of the process of study selection.

Four authors (Raphael Oberhuber (RO), Nikoletta Deluggi (ND), Regina Wiltsche (RW), Marina Blum (MB)) conducted the initial searches, and after initial exclusion at the title and abstract levels, 270 articles remained. These 270 articles were again independently screened at the title, abstract and text levels. The remaining 170 articles were then reviewed by two researchers [Johannes Hofer (JH) and MB] at the text level, which resulted in a final selection of 78 articles with 100% agreement. The reference lists of the selected articles were examined for the possibility of additional relevant studies; however, no additional studies were found. Figure 1 shows the flow diagram of the review process.

A standardized data extraction form was used to extract data from the included articles. For each article, the following data were extracted: (i) study title, (ii) names of authors, (iii) Digital Object Identifier, (iv) year of publication, (v) journal, (vi) details of the study population, (vii) number of participants, (viii) percentage of male participants, (ix) ethnicity of participants, (x) inclusion criteria, (xi) exclusion criteria, (xii) study type, (xiii) outcome measures, (xiv) methods/tests used, (xv) main findings of the study. The standardized neurodevelopmental measures extracted were categorized into four main domains: cognition, psychosocial functioning, language/communication/speech production and motor functioning. Table 1 lists the domains assigned to the set constructs.

**Table 1:** Domains and assigned constructs.

Domains	Cognitive	Speech-Language (incl. speech motor)	Psycho-social	Motor
Constructs:	Non-verbal development Visual perception Executive functioning Attention Memory Adaptive skills Social cognition	Vocabulary expressive Vocabulary receptive Grammar expressive Language receptive Gesture Use Social communication Narrative skills Speech-motor functions Intelligibility	Child's quality of life Emotion and Behavior Social functioning Mental health	Gross motor skills Fine motor skills Overall motor skills

## Results

Seventy-eight articles met the inclusion criteria of the study. The majority of these (57/78; 73%) were published in the United States, and the remaining 21 in other high-income countries. No study was conducted in a middle- or low-income country.

The publication years of the articles ranged from 1995 to 2021, with a median of 2013 (SD = 5.7). All studies were published within the past 28 years, and 60% in the last ten years.

Most studies (58%) used a retrospective, cross-sectional, or mixed study design, while the remaining 42% used a prospective and/or longitudinal design. The adapted NOS scores for the studies included ranged from 3 to 7 for a maximum of seven points, with a mean of 5.4. Table 2 lists all longitudinal studies included and all other studies with a NOS score >4. A total of 12.125 participants, ranging in age from 1 month to 15 years, were included across the articles. All extracted measures referred to the age group 1–8 years. The number of participants ranged from 10 to 1.770, with a mean of 155 (SD = 236.5). Thirteen different patient cohorts were found (Table 3). Forty-seven (59%) articles used data taken from these 13 cohorts.

**Table 2:** Summary of all included longitudinal studies and all studies with an adapted NOS score >4.

Ref. (#)	Design	*N*	Inclusion criteria	Exclusion criteria	Age (m)	Standardized ND measures	Cont.	Main findings	NOS
(26)	long	100	All children at NICU of the study site needing ≥1 cardiac surgical procedure prior to 8 months	Additional genetic diagnosis	6, 12–16, 24	BSDI-II, Revised Gesell Developmental Index gross motor quotient, Alberta Infant Motor Scale	N/A	Early motor outcomes worse in CHD with single ventricle physiology; high rate (43%–67%) of referral for early intervention services	5
(27)	long	106	infants < 3 months with CHD scheduled for repair using CPB	≥36 weeks gestation, congenital syndromes	0–24	BSID, PDMS, ASQ	N/A	Significant gross motor difficulties at 8 months Fine motor skills decrease from 8 to 24 months	5
(28)	long	10	CHD children <17.3y 3y after H/HL tx domiciled in UK/Ireland	N/A	15.6–183.6	GMDS, BAS, CBCL, Rutter B Scale, MFQ	N/A	33% behavior problems 12 months after tx and 75% 3 years after tx; prevalence of depression 23% at 12 months after tx and 13% at 3 years after tx	5
(29)	long	64	CHD children with high risk of developmental delay	Not speaking English	21–48	BSID-III, WPPSI full scale IQ, WRAVMA	N/A	many patients (20%–27%) who scored in the average range of BSID-III at age 2 showed deficits at age 4	6
(30)	long	293	The SVR trial and extension study followed neonates with CHD at 15 US centers for ≥6 years	N/A	36–72	HRQOL, BASC-2, Peds-QL, and FS-II(R)	N/A	Lower SES and less maternal education associated with greater early delays in communication and problem-solving	6
(31)	long	35	SV physiology born in or transferred to Toyama University Hospital between January 2002 and April 2012. 3 years of age at first testing.	Gestational age was less than 36 weeks or if they had genetic or malformation syndromes and history of CNS disease.	36–96	BSID II or III at 3 years and WISC 4 at 8 years.	HC	Weight at birth and stage II (second surgical stage: bilateral Glenn shunt) correlated with MDI; CHD children were more likely to require special education services.	6
(32)	long	68	School starters undergone at least one invasive procedure for correction/palliation of a major heart defect	Diagnosed ND syndromes	54–66	WPPSI, NEPSY, BSI (GSI), Maternal Worry Scale, Impact on Family Scale, CBCL	N/A	non-significant drop in the mean CBCL Total Problem Behavior Score in the Intervention group; no differences in school functioning,	6
(33)	long	99	CHD children with high risk of DD if undergone ≥3 evaluations in the follow-up clinic,	No exclusion based on race, language, or coexisting conditions	0–36	BSID III	N/A	Cognitive and language scores declining in subjects with syndromes but not in those without; Predictors of development: age, need for tube feeding, cardiopulmonary bypass time, time since last hospitalization	6
(34)	long	294	Tx-free survivors from SVR trial (HLHS or other related single right-ventricle abnormality and a Norwood procedure was planned)	cardiac anatomy that prohibited either the MBTS or the RVPAS or a major extracardiac abnormality that could independently affect the likelihood of transplant-free survival	36–72	BASC-2, ASQ; BSID	N/A	Children with Adaptive Skills Composite scores < 2 SD at the age of 6 months more likely to have DD at 14 months	6
(35)	ps	94	neonates with hypoplastic left heart syndrome after Norwood procedure	N/A	24	BSID-II	N/A	Mean PDI scores significantly higher and the incidence of psychomotor delay was significantly lower in the RVPA era	6
(36)	c/s	58	Children with CHD	BMI > 95th PC, body temperature >37.8°C, food allergies, dentofacial deformities, tooth loss from caries or trauma, dental pain, orthodontic and orthopedic treatment, any syndromes, neurological or cognitive changes, chronic use of medications, being under speech therapy, children not eating consistently	36–60	Montreal Children's Hospital Feeding Scale OMES-E, the Child language test (ABFW),	HC	CHD group displayed a significant increase in the likelihood speech alterations compared to the control. chance of speech impairment decreases as age advances	6
(37)	ps	109	Cases with prenatal diagnosis of complete ACC	No complete ACC	0–72	ADSI, WISC IV, Stanford Binet Intelligence Scale	N/A	Isolated complete ACC associated with a favorable outcome.	6
(38)	c/s	102	Children with CHD meeting the American Heart Association/American Academy of Pediatrics high-risk criteria for ND delay	not English-speaking; acquired cardiomyopathy;	51.6–56.4	WPPSI-III/IV, Woodcock Johnson III Test, VMI, WRAVMA, CCC-II, ABAS-II, BRIEF, Conners’ Parent Rating Scale-Revised-Short Form, CBCL, SRS	N/A	ND scores did not differ based on cardiac anatomy (1V vs 2V); both groups scored lower than norms on fine motor and adaptive behavior skills, children with genetic conditions scored worse	6
(39)	c/s	265	HLHS and other single right-ventricle anomalies; data from the Pediatric Heart Network SVR trial	N/A	60–72	ASQ, VABS, BASC-II, Peds-QL, CHQ-PF50, Functional Status II ®	N/A	Patients in the lowest SES tertile reported lower functional status and lower fine motor, problem-solving, adaptive behavior, and communication skills at 6 years	6
(40)	c/s	173	7–45 days old CHD infants with SV physiology with stable pulmonary and systemic blood flow predicted to undergo a SCPC	prematurity, SGA, systemic oxygen saturation <65%, creatinine >1.0 mg/dl, absolute neutrophil count <1,000 cells/ml, prior use of ACE inhibitor or other clinical situation preventing ACE inhibitor use	6–14	BSID	N/A	Higher BNP associated with impaired growth and poorer ND outcomes	6
(41)	rs	106	Patients Referred to the NICU CHD program January 01.01.2014 to 01.01.2015	<6 months age	0–36	standardized neurological exams, BSID III	N/A	53% below the ASQ-3 cutoff in the gross motor domain, 29% in the problem-solving domain, BSID-III scores <85 in 23%–41% of the population.	6
(42)	rs	329	Children with CHD and detailed postoperative temperature data, developmental evaluation at ages 1 years and/or 4 years	Any infant with a lowest rectal temperature greater than 18°C	12–48	BSID; WPPSI	N/A	ND outcome not significantly affected by the early postoperative body temperature profile of the infant	6
(43)	ps	31	patients requiring ECMO post-Norwood	<5 years	60–84	VABS-II PedsQL	matched surviving non- ECMO controls	VABS-II in normal range and comparable between ECMO-cases and non-ECMO control; the perceived physical appearance is lower among ECMO survivors by patient and proxy report.	6
(18)	rs	1,770	Children with CHD and a cardiac surgery using CPB at 9m, available BSID-I/II data at 6 months and 30 months of age	Children treated with a primary strategy of cardiac transplantation	10–18	BSID	N/A	PDIs and MDIs lower than normative means, risk factors for lower PDI: lower birth weight, white race, presence of a genetic/extracardiac anomaly; risk factors for lower MDI: lower birth weight, male gender, lower level of maternal education, presence of a genetic/extracardiac anomaly	6
(44)	ps	250	The SVR trial and extension study followed neonates with CHD at 15 US centers for ≥6 years; 6 years findings on Tx-free survivors	N/A	72	VABS-II, BASCII, PedsQl 4.0	N/A	At 6 years, children with HLHS show difficulties in areas of adaptive behavior, behavioral symptoms, QoL, and functional status. Risks for adverse outcomes: sociodemographic factors, measures of greater course complexity	6
(45)	ps	277	The SVR trial and extension study followed neonates with CHD at 15 US centers for ≥6 years; Findings on Tx-free survivors	cardiac anatomy prohibiting MBTS or RVPAS or an extracardiac abnormality affecting the likelihood of Tx-free survival	14–36	ASQ BSID-II	N/A	ASQ scores significantly lower than normal ASQ domain scores at 3 years; More complications, abnormal growth, and evidence of feeding, vision, or hearing problems independently associated with lower ASQ scores; Impaired ND at 3 years in children with single right-ventricle anomalies	6
(46)	c/s	105	Infants with CHD <8 weeks	Gestational age < 36 weeks, known syndrome or genetic anomaly associated with abnormal ND, ECMO prior to surgery	22–26	ITSEA, BSID-III	N/A	Delayed ND; Social-emotional outcome similar to Australian norms in all domains but better than the American-based norms in the Internalizing domain. Higher maternal education is associated with better ND outcomes and better scores in the internalizing and externalizing domains.	6
(47)	ps	61	1–41 months old children with cyanotic or hemodynamically impaired CHD	Children with history of hypoxic birth, prematurity, hypoglycemia, epilepsy, neurologic disease, or a genetic syndrome	1–41	BSID-III	HC	Patients showed significantly lower mean scores in all BSID-III subscales.	6
(48)	rs	53	<1 year at time of cannulation and receiving cardiac surgery for a CHD before initiation of ECMO	ECMO before surgical repair ECMO due to CDH	12–54	BSID, Stanford Binet Intelligence Scale, Mc-Carthy Scales of Children's Abilities, WISC	N/A	No survivor with an aortic cross-clamp time >40 min had a normal cognitive outcome	6
(49)	ps	35	Children with CHD 5–10 years after corrective surgery for TOF or VSD	N/A	60–120	oral and speech motor control function test (TFS), oral and speech apraxia test (Mayo Test)	N/A	Children with preoperative hypoxemia due to cyanotic defects at higher risk for dysfunction in speech/language than those with preoperative hypoxemia due to acyanotic defects.	6
(50)	c/s	24	All HLHS patients ≥3 years after at least two stages of the Norwood procedure	N/A	36–72	WPPSI-R, PPVT, Beery VMI; VABS; IQ testing	HC siblings first cousin	Median full-scale IQ and adaptive behavior scores patients: 88 and 91, family controls scored higher, significant differences in adaptive behavior	6
(51)	ps	94	Children with CHD needing early surgical correction	N/A	0–64.2	WPPSI, PPVT, PDMS, CBCL, VABS WeeFIM	N/A	Boys with CHD at enhanced risk for neuromotor impairments and activity limitations	6
(52)	c/s	93	Children with CHD and a Norwood–Sano repair at ≤6 weeks	N/A	18–24	BSID-III	N/A	No difference between HLHS and non-HLHS group for cognitive, language and motor scores; dominant right ventricle anatomy predictive of lower language and motor scores	6
(19)	c/s	90	≥1 invasive procedure for correction or palliation of a CHD	Children with developmental or psychiatric syndromes	48–61.2	NEPSY WPPSI-R.UK, CBCL; BSI, Maternal Worry Scale, Parenting Locus of Control Scale, Family Environment Scale, Significant Others Scale	children with mild heart defects	Compromised neuropsychological outcomes associated with a combination of cyanotic conditions and open-heart surgery; cyanotic and acyanotic conditions associated with sensorimotor delays; only children with complex conditions and palliative interventions at risk of poor behavioral outcomes; family processes predictors of behavioural outcomes	6
(53)	c/s	86	Children with CHD and an open-heart surgery and a birth weight >2,000 g,	Perinatal complications, noncardiac malformations, genetic abnormalities, physical or mental disorders	96	Short-form WISC-III NL NEPSY	HC sex, age and education matched	Objective and subjective measures of cognitive functioning are in agreement, indicating neurocognitive deficits in children with CHD	6
(54)	c/s	86	Postoperative Children with CHD at the age of 6–12 years	DiGeorge-velocardiofacial syndrome	96	NEPSY WISC III	HC	CHD children: mild motor and language deficits, Attention/EF and memory affected	6
(55)	ps	233	Children with CPB surgery between 2004 and 2009	died before 6 years examination -aged ≥6 years at the time of surgery -undergone CPB surgery before enrollment	61.2–81.6	WPPSI-III, SON, K-ABC, WISC-III, BSID-II, Zurich Play Behavior, Zurich Neuromotor Assessment	N/A	Lower cognitive and motor performance after CBP; predictors for impaired ND: genetic disorder, longer length of intensive care stay, lower birth weight, postoperative seizures, and lower SES.	6
(56)	c/s	334	Infants with complex CHD and heart surgery at 6w requiring CPB but not ECMO or HT prior to 21 ± 3 months assessment	Children who died prior to assessment and those lost to follow-up No BSID-III	0–24	BSID III ABAS-II	N/A	GTF group 8 times the number of children delayed on the general adaptive composite score. Independent OR for GTF: presence of a chromosomal abnormality, SV anatomy, total postoperative days of open sternum, total number of hospital days	6
(57)	ps	980	< 1 years at enrollment, 9–18 months at follow up, living in US	N/A	9–18	BSID III	HC	association between moderate to severe hypoglycemia and poorer 1 year ND outcomes	6
(58)	ps	43	Children with HLHS or other forms of UVH	Cantrell's pentalogy chromosomal defect	12.2	The GMDS and Alberta Infant Motor Scale	HC	Lower mean GMDS quotient and Alberta Infant Motor Scale scores in children with HLHS. predictors of low developmental quotient: HLHS, clinical history of seizure and high plasma lactate levels after the bidirectional Glenn operation.	6
(59)	c/s	67	infants with CHD admitted to the NICU from 2005 to 2013	chromosomal abnormalities, not operated, no Bayley III evaluation	36	Bayley-III	67 VLBW infants +81 HC	CHD children and VLBW controls with significant deficits in language, cognition, and motor skills scores; SV infants lowest scores for language and gross motor	6
(60)	c/s	51	All Fontan survivors invited to participate at the age of 34–96m	Internal pacemaker wires in place or if the parents declined	34–96	WISC, MRI	HC	HLHS group significantly lower WISC scores than the non-HLHS subgroup, neither subgroup scored significantly differently from the standard population. Predictors for ND outcome: SES, circulatory arrest, perioperative seizures	7
(61)	rs	52	children at two years of age with single-ventricle CHD	No genetic comorbidities	24	MRI and BSID-III	HC	Brain volumes smaller in patients compared with controls, CSF volumes were greater. CSF volume associated with ND outcome, accounting for 21% of variability in the cognitive composite score.	7
(62)	c/s	77	Children with HLHS or UVH born between 08/2002 and 02/2005	N/A	30	BSID II, MacArthur Communicative Development Inventories, and CBCL	HC	Mean MDI within average range but significantly lower than HC. HLHS children greater delays in expressive language scores; 35% of the children with HLHS performed in the lowest 10%	7
(63)	ps	144	Children with CHD at the age of 1y whose parents could read German fluently	N/A	12–48	TAPQOL—a standardized questionnaire on child HRQoL at 1 year and 4 years of age	HC	HRQoL of infants and preschool-age children with CHD is impaired in physical, motor and cognitive dimensions.	7

ABAS, adaptive behavior assessment system; ABFW, child language test; ACC, agenesis of the corpus callosum; ADSI, ankara developmental screening inventory; ASQ, ages and stages questionnaire; BAS II, British ability scales II; BASC, behavior assessment system for children; BRIEF, behavior rating inventory of executive function-preschool; Beery-VMI, Beery-Buktenica developmental test of visual-motor integration; BMI, body mass index; BNP, B-type natriuretic peptide measurements; BSI, brief symptom inventory; BSID, bayley scale of infant development; Conners, Conners' parent rating scale-revised-short form; CBCL, child behavior checklist; CCC-II, children's communication checklist; CDH, congenital diaphragmatic hernia; CHD, congenital heart defect; CHQ-PF50, child health questionnaire 50 items; CNS, central nervous system; CPB, cardiopulmonary bypass; CSF, CerebroSpinal fluid; c/s, cross-sectional; DD, delayed development; ECMO, ExtraCorporeal membrane oxygenation; EF, executive functions; FS-II (R), functional status II ®; GMDS, functional status II ®; GSI, general severity index; GTF, gastrostomy tube feeding; HC, healthy control; HLHS, hypoplastic left heart syndrome; HRQoL, health-related quality of life; HT, heart transplantation; ITSEA, infant-toddler social and emotional assessment; K-ABC, Kaufman assessment battery for children; MBTS, modified blalock-taussig shunt; MRI, magnetic resonance imaging; ND, neurodevelopmental; NICU, Neonatal Intensive Care Unit; NEPSY, a developmental NEuroPSYchological assessment; OMES-E, orofacial myofunctional assessment protocol; OR, odds ratio; PedsQL, pediatric quality of life inventory; SES, socio-economic status; PDI, psychomotor development index; MDI, mental development index; MFQ, mood and feelings questionnaire; long, longitudinal; Peds-QL, pediatric quality of life inventory; ps, prospective; PDMS, peabody developmental motor scale; PPVT, peabody picture vocabulary test; rs, retrospective; RVPAS, right ventricle-to-pulmonary artery shunt; SCPC, superior CavoPulmonary connection; SV Physiology, single ventricle; SGA, small for gestational AGE; SON, Snijders-Omen nonverbal test of intelligence; SRS, social responsiveness scale; SVR, pediatric heart network single ventricle reconstruction trial; TAPQOL, Netherlands Organisation for Applied Scientific Research Academic Medical Centre Preschool Children Quality of Life; Tx, transplant; TFS, total functional score; TOF, tetralogy Of fallot; UVH, univentricular heart; VLBW, very-low birth weight; VSD, ventricular septal defect; VABS, vineland adaptive behavior scales; WISC, Wechsler intelligence scale; WISC, Wechsler intelligence scale for children; WRAVMA pegboard, wide range assessment of visual motor abilities; WPPSI, Wechsler preschool and primary scale of intelligence; WeeFIM, functional independence measure for children.

**Table 3:** Summary of CHD cohorts.

Cohort	Articles (*n*)	Articles #
Four provinces in Western Canada	6	(35, 52, 56, 64–66)
Ghent University Hospital	2	(53, 54)
The Children's Hospital of Philadelphia	4	(67–70)
Pediatric Heart Network Single Ventricle Reconstruction (SVR) trial	7	(30, 34, 39, 44, 45, 60, 71)
Herma Heart Center Developmental Follow-up Clinic (HHCDC)	4	(21, 29, 33, 38)
University of California, San Francisco	2	(48, 72)
University of Michigan	3	(43, 57, 60)
Toyama University Hospital	2	(31, 59)
University Children's Hospital Zurich	6	(55, 61, 63, 73–75)
Hospital for Sick Children in Toronto, Canada	2	(27, 76)
Clinique d'Investigation Neuro-Cardiaque	2	(77, 78)
Sainte-Justine University Hospital Centre.	6	(46, 58, 62, 79–81)
ISV trial	2	(82, 83)

Overall, eight longitudinal cohorts were included in the articles reviewed, with follow-ups conducted after 1–5 years.

The neurodevelopmental domains measured were distributed as follows: Cognition-associated constructs (53%) formed the main domain tested, followed by speech/language/communication (18%), psychosocial functioning (16%) and motor development (13%). Overall, a variety of standardized measures were used to describe neurodevelopmental functioning; however, the Bayley scales most prominent.

Tables 4A–C lists all extracted measures that were used in three or more publications according to domain, construct and target age group.

**Table 4:** (**A**)–(**C**) extracted standardized measures used in a minimum of 3 of the extracted publications.

(A)	12–14 months	
Measures	Constructs	Number of studies
BSID III Cognitive scale	Non-verbal Development/Cognition, Visual Perception	24
BSID III Adaptive Behavior scale	Social Cognition, Adaptive Skills	23
BSID III Social Emotional scale	Social Cognition	23
BSID II- MDI	Social Cognition, Non-verbal Development/Cognition, Adaptive Skills	19
WPPSI (III, IV, R: UK, R)	Non-verbal Development/Cognition, Visual Perception, Executive functioning, Memory	17
Vineland (I, II, III)	Social Cognition, Adaptive Skills	9
ABAS-(I, II)	Adaptive Skills	4
ASQ	Social Cognition, Adaptive Skills	4
Beery-VMI (I, V)	Visual Perception	4
NEPSY—complete	Non-verbal Development/Cognition, Visual Perception, Executive functioning, Attention, Memory	4
WJ-III reading and math clusters	Non-verbal Development/Cognition	4
KABC (I, II)	Non-verbal Development/Cognition, Memory	3
MSCA	Non-verbal Development/Cognition, Visual Perception, Executive functioning, Attention, Memory	3
NEPSY—Attention/EF CDS	Executive functioning, Attention, Memory	3
BSID III Motor scale	Fine Motor Skills, Gross motor Skills	23
BSID II—PDI	Fine Motor Skills, Gross motor Skills	18
Vineland (I, II, III)	Overall motor skills	9
WRAVMA	Fine Motor Skills	5
ASQ	Overall motor skills, Fine Motor Skills, Gross motor Skills	4
Beery-VMI (I, V)	Overall motor skills, Fine Motor Skills, Gross motor Skills	4
NEPSY complete	Fine Motor Skills	4
MSCA	Overall motor skills	3
PedsQL 4.0	Physical health	3
BSID III Language scale	Vocabulary Expressive, Vocabulary Receptive, Grammar expressive, Language receptive	24
BSID III Adaptive Behavior scale	Social communication	23
WPPSI (III, IV, R: UK, R)	Vocabulary Receptive, Language receptive	17
Vineland (I, II, III)	Social communication	9
WPPSI-III FSIQ	Vocabulary Receptive, Language receptive	9
ASQ	Social communication, Narrative skills	4
NEPSY—complete	Grammar expressive, Language receptive, Narrative skills	4
WJ-III reading and math clusters	Vocabulary Expressive, Vocabulary Receptive, Grammar expressive, Language receptive, Narrative skills	4
BSID III Social Emotional scale	Social Functioning, Emotion and behavior	23
BSID III Adaptive Behavior scale	Social Functioning, Emotion and behavior	23
Vineland (I, II, III)	Social Functioning	9
ASQ	Social Functioning	4
BASC-II	Emotion and behavior, Mental health	4
FS-II (R)	Social Functioning	4
NEPSY—complete	Social Functioning	3
PedsQL 4.0	Social Functioning, Child QoL, Emotion and behavior, Mental health	3

(B)	42–60 months	
Measurements	Constructs	Number of studies
BSID III Cognitive scale	Non-verbal Development/Cognition, Visual Perception	24
BSID III Social Emotional scale	Social Cognition	23
BSID III Adaptive Behavior scale	Social Cognition, Adaptive Skills	23
WPPSI (III, IV, R: UK, R)	Non-verbal Development/Cognition, Visual Perception, Executive functioning, Memory	17
Vineland (I, II, III)	Social Cognition, Adaptive Skills	9
BAS (I, II)	Executive functioning, Attention, Memory, Adaptive Skills	7
ABAS-(I, II)	Adaptive Skills	4
ASQ	Social Cognition, Adaptive Skills	4
Beery-VMI (I, V)	Visual Perception	4
NEPSY—complete	Non-verbal Development/Cognition, Visual Perception, Executive functioning, Attention, Memory	4
WJ-III reading and math clusters	Non-verbal Development/Cognition	4
KABC (I, II)	Non-verbal Development/Cognition, Memory	3
MSCA	Non-verbal Development/Cognition, Visual Perception, Executive functioning, Attention, Memory	3
NEPSY—Attention/EF CDS	Executive functioning, Attention, Memory	3
BSID III Motor scale	Fine Motor Skills, Gross motor Skills	23
Vineland (I, II, III)	Overall motor skills	9
WRAVMA	Fine Motor Skills	5
ASQ	Overall motor skills, Fine Motor Skills, Gross motor Skills	4
Beery-VMI (I, V)	Overall motor skills, Fine Motor Skills, Gross motor Skills	4
NEPSY complete	Fine Motor Skills	4
MSCA	Overall motor skills	3
PedsQL 4.0	Physical health	3
BSID III Language scale	Vocabulary Expressive, Vocabulary Receptive, Grammar expressive, Language receptive	24
BSID III Adaptive Behavior scale	Social communication	23
WPPSI (III, IV, R: UK, R)	Vocabulary Receptive, Language receptive	17
Vineland (I, II, III)	Social communication	9
WPPSI-III FSIQ	Vocabulary Receptive, Language receptive	9
ASQ	Social communication, Narrative skills	4
NEPSY—complete	Grammar expressive, Language receptive, Narrative skills	4
WJ-III reading and math clusters	Vocabulary Expressive, Vocabulary Receptive, Grammar expressive, Language receptive, Narrative skills	4
BSID III Social Emotional scale	Social Functioning, Emotion and behavior	23
BSID III Adaptive Behavior scale	Social Functioning, Emotion and behavior	23
Vineland (I, II, III)	Social Functioning	9
ASQ	Social Functioning	4
BASC-II	Emotion and behavior, Mental health	4
FS-II (R)	Social Functioning	4
BAS-II	Social Functioning, Emotion and behavior	3
NEPSY—complete	Social Functioning	3
PedsQL 4.0	Social Functioning, Child QoL, Emotion and behavior, Mental health	3

(C)	72–96 months	
Measurements	Constructs	Number of Studies
WPPSI (III, IV, R: UK, R)	Non-verbal Development/Cognition, Visual Perception, Executive functioning, Memory	17
Vineland (I, II, III)	Social Cognition, Adaptive Skills	9
BAS (I, II)	Executive functioning, Attention, Memory, Adaptive Skills	7
Beery-VMI-V	Visual Perception	4
NEPSY—complete	Non-verbal Development/Cognition, Visual Perception, Executive functioning, Attention, Memory	4
WJ-III reading and math clusters	Non-verbal Development/Cognition	4
KABC (I, II)	Non-verbal Development/Cognition, Memory	3
MSCA	Non-verbal Development/Cognition, Visual Perception, Executive functioning, Attention, Memory	3
NEPSY—Attention/EF CDS	Executive functioning, Attention, Memory	3
Vineland (I, II, III)	Overall motor skills	9
WRAVMA	Fine Motor Skills	5
Beery-VMI-V	Overall motor skills, Fine Motor Skills, Gross motor Skills	4
NEPSY complete	Fine Motor Skills	4
MSCA	Overall motor skills	3
PedsQL 4.0	Physical health	3
WPPSI (III, IV, R: UK, R)	Vocabulary Receptive, Language receptive	17
Vineland (I, II, III)	Social communication	9
WPPSI-III FSIQ	Vocabulary Receptive, Language receptive	9
NEPSY—complete	Grammar expressive, Language receptive, Narrative skills	4
WJ-III reading and math clusters	Vocabulary Expressive, Vocabulary Receptive, Grammar expressive, Language receptive, Narrative skills	4
Vineland (I, II, III)	Social Functioning	9
BASC-II	Emotion and behavior, Mental health	4
FS-II (R)	Social Functioning	4
BAS-II	Social Functioning, Emotion and behavior	3
NEPSY—complete	Social Functioning	3
PedsQL 4.0	Social Functioning, Child QoL, Emotion and behavior, Mental health	3

ABAS, adaptive behavior assessment system; ASQ, ages and stages questionnaire; BAS II, British ability scales II; BASC, behavior assessment system for children; BSID, Bayley scales of infant development; Beery-VMI, Beery-Buktenica developmental test of visual-motor integration; FSIQ, full-scale IQ score; FS-II (R), functional status II ®; K-ABC, Kaufman assessment battery for Children; MSCA, McCarthy scales of children's abilities, MDI, mental development index; MBCDI, MacArthur-Bates communicative development inventories; NEPSY, developmental NEuroPSYchological assessment; PedsQL, pediatric quality of life inventory; PDI, psychomotor development index; VABS, Vineland adaptive behavior scales; WJ-III reading and math clusters, Woodcock-Johnson IIIreading and math cluster; WPPSI, Wechsler preschool and primary scale of intelligence; WRAVMA pegboard, wide range assessment of visual motor abilities.

The extracted measures are grouped according to three age groups: a) 12–42 months, b) 42–60 months and c) 72–96 months. The constructs measured are listed, as are the numbers of studies using each measure. The colors correspond to the domains of the given measure with: orange = cognitive, gray = motor, blue = speech/language, violet = psycho-social. Measures with font color red indicate questionnaires and interviews. All other measures represent direct assessments.

Thirty-nine of the measures (54%) were used in only one study. Thirty-nine (50%) of the 78 articles excluded children with syndromes, and nine (12%) excluded children with cognitive disabilities.

## Discussion

The goal of this review was to generate a concise overview of the methodology used to document neurodevelopment in young children aged 1–8 years with CHD, identify factors that are modifiable by intervention and to identify research gaps that need to be addressed by future studies.

Seventy-eight studies met the inclusion criteria, all of which were conducted in high-income countries within the past 28 years. That no study from a low- or middle-income country was found demonstrates the compelling need to extend rigorous science and innovative clinical practice by focusing on stepped care processes at the global level.

The quality of the studies included, assessed by means of an adapted Newcastle-Ottawa Scale, ranged from fair to excellent. Most studies (58%) used a retrospective, cross-sectional, or mixed study design, while the remaining 42% used a prospective and/or longitudinal design. Less than half of the studies (30/78, 38%) included representative samples that did not exclude severely affected children (28–31, 33–35, 37–45, 48–52, 56, 60–63, 74, 79, 82, 84, 85). Notably, 50% of the 78 articles excluded children with various syndromes, and 12% excluded children with cognitive disabilities. This is especially true for the few larger longitudinal studies (26, 27): Children with chromosomal changes and/or additional impairments were excluded. Insufficient recruitment of children with poor cardiac outcomes and disproportionate inclusion of privileged children and families are common. Including children with special needs requires additional time and knowledge. It is challenging to combine age-appropriate testing with testing that is appropriate to the individual child's level of development. This finding is not surprising and is well known for other well-studied patient cohorts, for instance, children with hearing loss (86) and children with autism spectrum disorders (87). Epidemiological study designs are needed to ensure that all children with CHD are included in our understanding of neurodevelopment and neurodevelopmental trajectories and their potential malleability.

Longitudinal data on the neurodevelopment of children with CHD are restricted to 8 different study cohorts, where follow-ups were conducted over a maximum of five years. Despite their high quality and their scene-setting impact, these studies did not use cohorts that were broadly representative. Considering the recommendations from the Cardiac Neurodevelopmental Outcome Collaborative (22), the measures chosen for neurodevelopmental trajectories were not sufficiently fine-tuned to allow malleable predictors of good neurodevelopmental outcomes to be identified.

Overall, trajectories of structural language development, especially social communication and speech production, have been less researched than other domains of cognitive functioning. With increasing age of the children studied, published data on (i) language and communication and (ii) speech motor and motor function decrease, and neurodevelopmental measures within the cognitive domain become even more predominant.

Considering the predominant use of the Bayley Scales for early infant development and the current literature pointing towards an overestimation of neurodevelopment using the Bayley III scales and possible underestimation by using the Bayley II scales (88–92), there is a need for supplementary measures of early childhood neurodevelopment. However, the Bayley III language scales provide a good estimate of language development (88, 91, 92).

There remains a lack of research into measures of language and communication early in life and into everyday use of language, although social communication is expected to impact language development, social cognition, peer interaction, and mental health later in life. In addition, standardized measures of child self-regulation and parent-child interaction are missing and need to be addressed in future studies, as they represent important, potentially malleable predictors.

Overall, a variety of standardized measures have been used to describe neurodevelopmental functioning, with the Bayley scales dominating. Comparing the results of this scoping review to the recent recommendations for neurodevelopmental assessment (22, 23) highlights the importance of a clear and standardized guidance for neurodevelopmental assessments.

Given the known risks associated with CHD and the demonstrated benefit of early intervention in other populations (93–96), regular monitoring and periodic neurodevelopmental assessment are critical throughout childhood in order to optimize the neurodevelopmental outcomes of patients with CHD.

Standard application of well-balanced neurodevelopmental assessment batteries across cardiac neurodevelopmental sites holds enormous promise for both clinical care and research within the CHD population.

The findings discussed above must be considered in the light of specific limitations of this scoping review and the limitations of scoping reviews in general:

Including studies only by searching the electronic database PubMed may exclude further relevant published literature and grey literature, which leads to potential bias in the findings. This can result in an incomplete representation of the evidence available. The used search terms lack infant specific formulations like regulation or attention.

In conclusion, our systematic review of the neurodevelopmental assessment tools used in children with complex CHD identified the following research gaps:

➢ No data on low- or middle-income countries,
➢ There is a lack of representative studies of the whole cohort of children with severe CHD: children with syndromes or intellectual disability are in most cases excluded,
➢ Need for longitudinal studies that focus on a balanced use of measures for all important neurodevelopmental domains,
➢ Data on social communication, expressive and receptive language, speech motor, and motor function are underrepresented,
➢ Presently, there remains a lack of (i) research into the everyday use of language and language and communication measures early in life and (ii) tools to measure early social communication skills.

## Data Availability

The original contributions presented in the study are included in the article/Supplementary Material, further inquiries can be directed to the corresponding author.
